# Clinical evaluation of tissue stops on 3D-printed custom trays

**DOI:** 10.1038/s41598-018-37826-7

**Published:** 2019-02-12

**Authors:** Kehui Deng, Hu Chen, Rong Li, Linlin Li, Yong Wang, Yongsheng Zhou, Yuchun Sun

**Affiliations:** 1 Center of Digital Dentistry, Peking University School and Hospital of Stomatology, Beijing, PR China; 2 Department of Prosthodontics, Peking University School and Hospital of Stomatology, Beijing, PR China; 3 National Engineering Laboratory for Digital and Material Technology of Stomatology, Beijing, PR China; 4 Research Center of Engineering and Technology for Digital Dentistry, Ministry of Health, Beijing, PR China; 5 Beijing Key Laboratory of Digital Stomatology, 22 Zhongguancun Avenue South, Haidian District, Beijing, 100081 PR China

## Abstract

This study evaluated the quality of impressions taken using three-dimensional (3D)-printed custom trays with different tissue stops to optimize the custom tray designs. Different custom trays were designed and printed based on six edentulous patients. These trays were divided into four groups based on the tissue-stop designs: 3DP trays (3D-printed trays without tissue stops), 3DPS trays (3D-printed trays with saddle-shaped tissue stops), 3DPM trays (3D-printed trays with marginal-band tissue stops) and 3DPIM trays (3D-printed trays with inner marginal-band tissue stops). Final impressions were taken using these trays, of which, the 3DP and 3DPIM trays were preborder-moulded. The finished complete dentures were used to take impressions that were set as the reference group to analyse the accuracy of the final impressions. The impressions taken using the 3DP custom trays (preborder-moulded) were used as a reference to analyse the extensions of the impressions taken using the other three custom trays. Randomized block or Friedman tests were used to evaluate each group’s statistical significance. The results revealed that the 3DPIM custom trays with the inner marginal-band tissue stop facilitated the preborder-moulding process and improved the accuracy and extension of the impression.

## Introduction

Accurate impressions provide the foundation for complete denture restoration and are closely related to the dentures’ retention and stability^1^. The precise morphology of the tissue surface and the appropriate extension range are key factors of the impression. Currently, a secondary impression method is most often used to take impressions with better morphology and quality^2–4^. However, when fabricating custom trays, the inevitable deformation of wax paving onto the cast often results in a nonuniform space for the final impression material^5–7^.

In a previous study, our research group explored a digital custom tray system^8^ in which the tray was designed using software and manufactured by a three dimensional (3D) printer. Experimental evaluations showed that these custom trays achieved greater accuracy with an appropriate extension range and a more uniform three-dimensional space for impression materials than those of traditional manual custom trays. However, the positions of both custom trays are difficult to accurately determine when they are covered with impression material to take the final impression in the patient’s mouth. Consequently, the impression material thickness can be nonuniform when taking the impression, which can lead to uncontrollable deformation of the alveolar mucosa, especially when the dentist is inexperienced.

Border moulding is important for obtaining an appropriate extension range and promoting complete denture retention^9,10^. During this process, with good plasticity of the impression compound, the dentist must keep the tray in place and functionally move the soft tissue around the alveolar ridge to determine the impression margin’s correct location and morphology, thereby forming the border seal of the custom tray at the margin. However, dentists often have difficulty precisely controlling the thickness and uniformity of the border-moulding material. They can only visually assess the border-moulding morphology to make adaptations, and even experienced dentists cannot always ensure the uniform thickness of the border-moulding material margins in a custom tray.

Tissue stop is another significant application that ensures precise custom tray placement^11–13^. Our group recently found that custom trays designed with saddle-shaped tissue stops can better control the impression material thickness but cannot promote formation of the impression’s margin morphology^14^. Thus, to improve the border moulding efficiency and quality, the custom trays’ structural design must be improved. In this study, we designed three tissue stops and identified the one that best obtained precise impressions. The results were evaluated using the impression surface precision and extension range.

## Materials and Methods

### Patient enrolment

This study was approved by the Bioethics Committee of Peking University School and Hospital of Stomatology (Beijing, China; approval number, PKUSSIRB-201627042). The procedures and risks involved with participating in this study were discussed with the volunteers, and written informed consent was obtained from each included participant. The methods were conducted in accordance with the approved guidelines.

Six edentulous patients from the Department of Prosthodontics of Peking University Hospital of Stomatology were enrolled. The patients had undergone complete tooth extractions 3 months prior. The inclusion criteria were as follows: maxillary and mandibular edentulous jaws; alveolar ridge absorption grading Atwood levels I–III; acceptance of complete denture restoration; and willingness to cooperate. The exclusion criteria were as follows: the presence of mental illness or Parkinson’s disease; inability to care for themselves; allergy to the denture material; obvious defects in the upper or lower jaw; severe oral mucosal diseases without effective treatment; an obvious flabby alveolar ridge; or a sensitive pharynx reflex. All denture restoration expenses were exempted to compensate the patients participating in the study. The experimental design and scheme were submitted to the Ethics Committee of Peking University Hospital of Stomatology for examination and were conducted after approval.

### Primary impression

Primary impressions were taken using stock trays with an impression compound and alginate. These procedures were performed by a dentist with more than 3 years of clinical experience. The acquired primary impressions were scanned into a 3D scanner (Smart Optics 880 Dental Scanner, Smart Optics, Germany).

### Digital design and 3D custom tray printing

**Figure 1:**
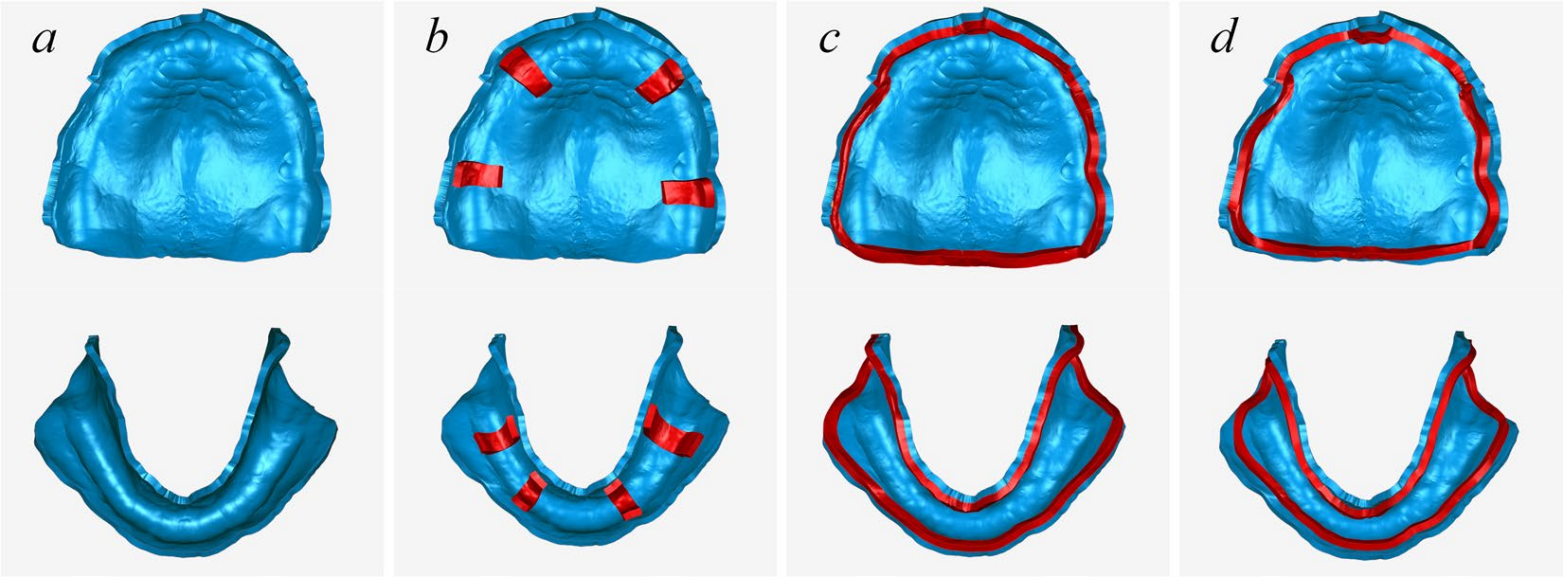
Tissue stop on the computer-designed custom tray: (**a**) no tissue stop, (**b**) saddle-shaped tissue stop, (**c**) marginal-band tissue stop, and (**d**) inner marginal-band tissue stop.

Scanned data from the impressions were imported into reverse engineering software (Geomagic Studio 2012, Raindrop Geomagic, USA) to design the custom trays. First, using an interactive plotted command, the tray area margin was extracted and trimmed. For the maxillary jaw, the labial/buccal margin was determined by the labial/buccal frenum and vestibular mucosa rugae, while the posterior margin was determined by the hamular notch and vibrate line (2 mm behind the fovea palatinae). For the mandibular jaw, the labial/buccal margin was determined by the labial/buccal frenum and vestibular mucosa rugae; the posterior area was confirmed to be covering the retromolar pad, and the lingual margin was determined by the lingual frenum, the mucosa rugae of the mouth floor and the retromylohyoid fossa. The surface data from the impression were trimmed, and the normal direction was reversed, meaning that the matrix surface was translated into the patrix surface. The trimmed surface was then offset along its new normal direction by 2 mm, forming the custom tray’s interior surface. To create the solid 3D model of the custom tray body, a 2-mm shell of the tray’s interior surface was drawn. Next, 3 tissue stops were designed: a saddle-shaped support (four tissue stops placed on the canines and first molar areas with sizes of 3 mm * 4 mm and a thickness of 2 mm), a marginal-band support (a band around the margin of the custom tray’s outer line with a width of 1–2 mm and a thickness of 2 mm), and an inner marginal-band support (similar to the marginal-band support but was placed approximately 1.5 mm farther inward than the marginal-band support to facilitate attaching the border-moulding wax). The tissue stops and custom tray bodies were fused into one object, and an appropriate handle was designed and connected (Fig. 1). The designed custom tray data were then saved in STL file format and printed using a fused deposition modelling (FDM) 3D printer (Lingtong I, BeijingSHINO, China) with polylactic acid (PLA) material, in which the layer height was set at 0.1 mm. The four trays were named 3DP trays (3D-printed trays without tissue stops), 3DPS trays (3D-printed trays with saddle-shaped tissue stops), 3DPM trays (3D-printed trays with marginal-band tissue stops) and 3DPIM trays (3D-printed trays with inner marginal-band tissue stops).

### Clinical verification

Final impressions for each edentulous patient were taken repeatedly with the four custom trays using alginate as the impression material, of which, the 3DP and 3DPIM trays were preborder-moulded before taking the impressions. The impressions were completed by a dentist with more than 3 years of edentulous restoration experience. To ensure that the experimental conditions remained consistent, each patient’s final impressions were completed by the same dentist within half a day. Each patient rested for half an hour between impressions to allow the soft tissue deformation to recover. Data from the final impressions were obtained using the same 3D scanner. The final complete denture, which was required to be worn for 2 weeks without tenderness and with good retention, mastication and pronunciation, was used as a tray to take the impression with the silicone rubber. These impressions acted as the reference data to evaluate the impressions made by the custom trays with different tissue stops.

#### Measurement of the impression precision

**Figure 2:**
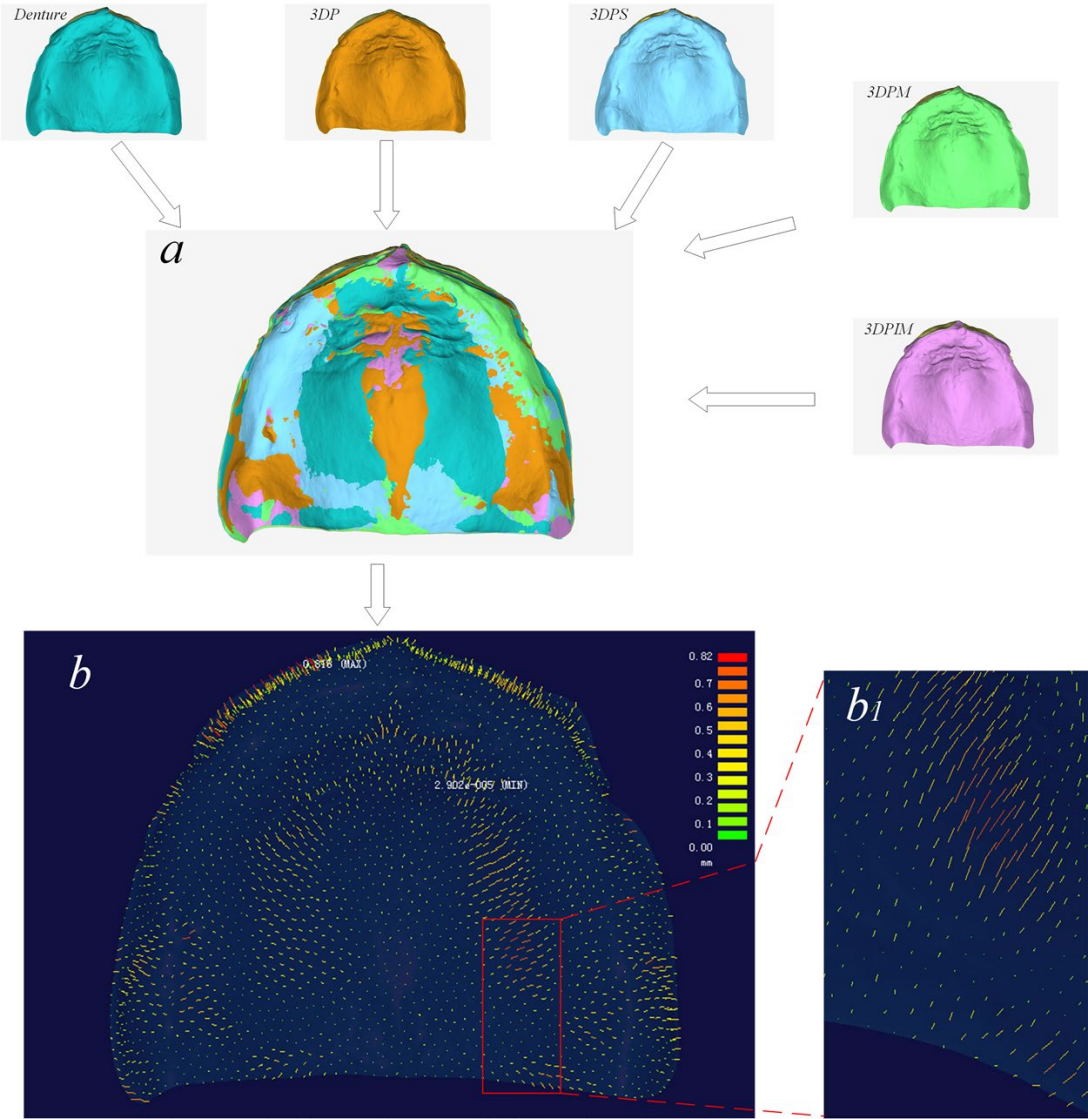
Analysis of the impression accuracy: (**a**) reference impression data “Denture” was registered with the data for the other four impressions and (**b**) the distance from the selected point to the reference surface was calculated; b1 is the partial enlargement map in graph b.

The cast perfused by the impression from the 3DP custom tray was used to fabricate a complete denture. After fabrication, the denture was ground appropriately in the patient’s mouth. When the patient returned after the 2-week trial without tenderness, the complete denture was used as the tray to obtain the functional impression. Patients were instructed to bite after the impression material covered the tissue surface of the denture placed in the patient’s mouth. The impression was scanned (named Denture) for reference to evaluate the accuracy of the impressions taken with the four custom trays in this experiment (Fig. 2). With registration commands in Geomagic software, the scanned data from the impression taken with the four custom trays were registered to the reference data, where the measured point cloud data were selected and simplified with a 0.5-mm interval. The distance from each point in the simplified point cloud to the triangle mesh surface of the reference impression data was measured using reverse engineering software (ImageWare 13.0, EDS, USA). These distance data were calculated using the root mean square value (RMS) to present the tissue surface deviation of the final impression.

1 $${\rm{RMS}}=\sqrt{\frac{{\sum }_{i=1}^{n}{x}_{i}^{2}}{n}}$$

In this case, n represents the number of measurement points, and ${x}_{i}$ represents the i^th^ measured value. This method was used to measure the three-dimensional morphological deviations of the impression surfaces from the four custom trays from one patient.

#### Extension of the impression

**Figure 3:**
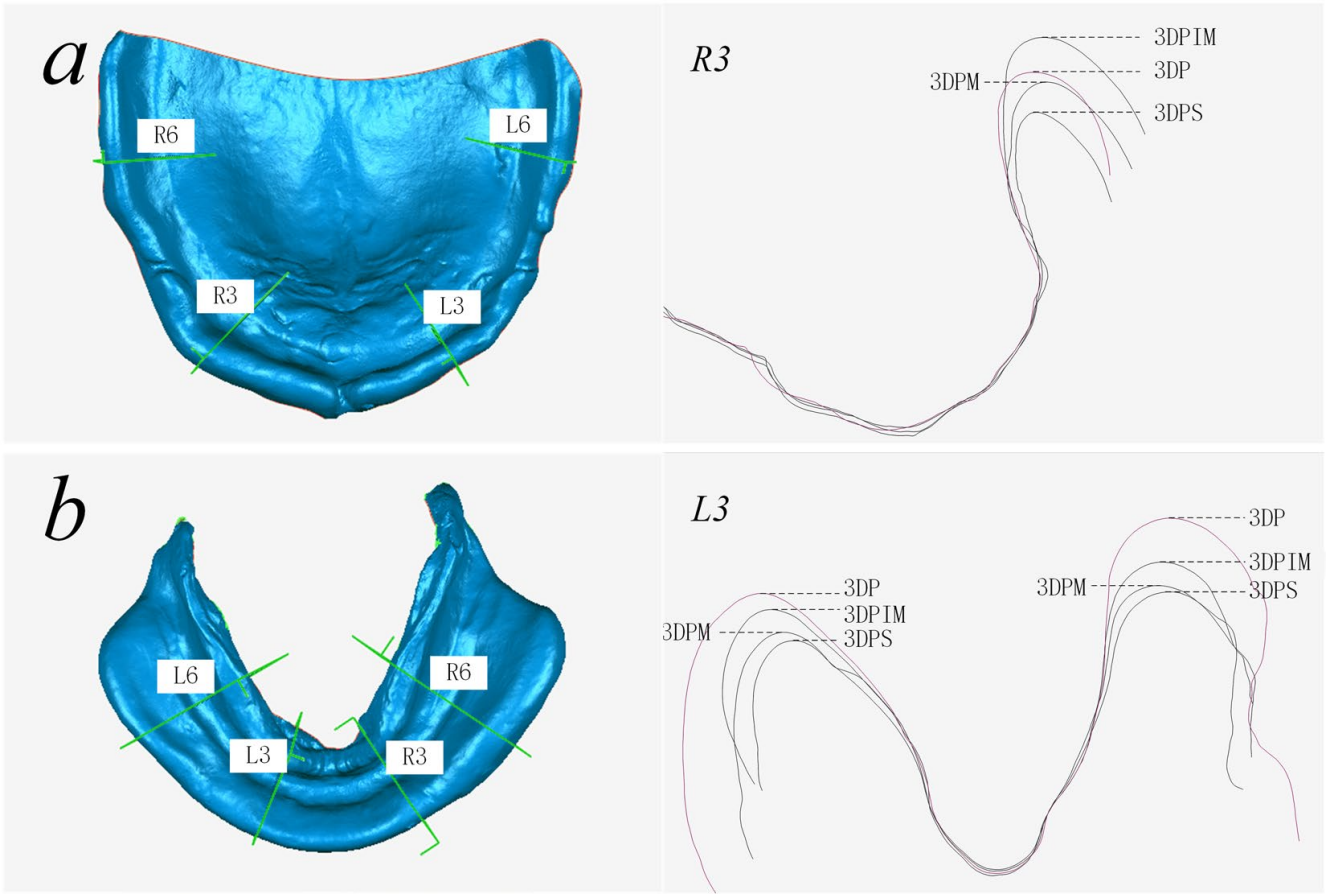
Evaluation of the margin extensions of the impressions: the upper and lower impression data were segmented longitudinally on the canines and the first molar position. The distance of the impression margin between the test and the reference data in the longitudinal view was measured: (**a**) longitudinal section sites of the maxillary impression, the profile at the R3 position; and (**b**) longitudinal section sites of the mandibular impression, the profile at the L3 position. L3 = left canine, R3 = right canine, L6 = left first molar, R6 = right first molar.

To evaluate the impression extensions, the final impression data from the four trays were imported into three-dimensional measurement and analysis software (Geomagic Qualify 12, Raindrop, USA), and the impression data from the 3DP custom trays were used as reference data. A buccolingual profile view on the sites of the canine and first premolar was established vertical to the occlusal surface (Fig. 3) to measure the distance in margin position between each impression and the reference impression. Positive values indicated that the extension of the test impression was smaller than that of the reference impression, while negative values implied the opposite.

### Statistical analysis

The mean and standard deviation of all measured data including the values of the impression precision and extension were analysed using statistical software (IBM SPSS Statistics v20.0, IBM, USA). The dataset was separated into two sections (upper jaw and lower jaw). Before comparing the differences between groups, the data were tested for normality. If the data were normal, a randomized block test was used to evaluate each group’s statistical significance; otherwise, a Friedman test was employed (α = 0.05).

## Results

**Figure 4:**
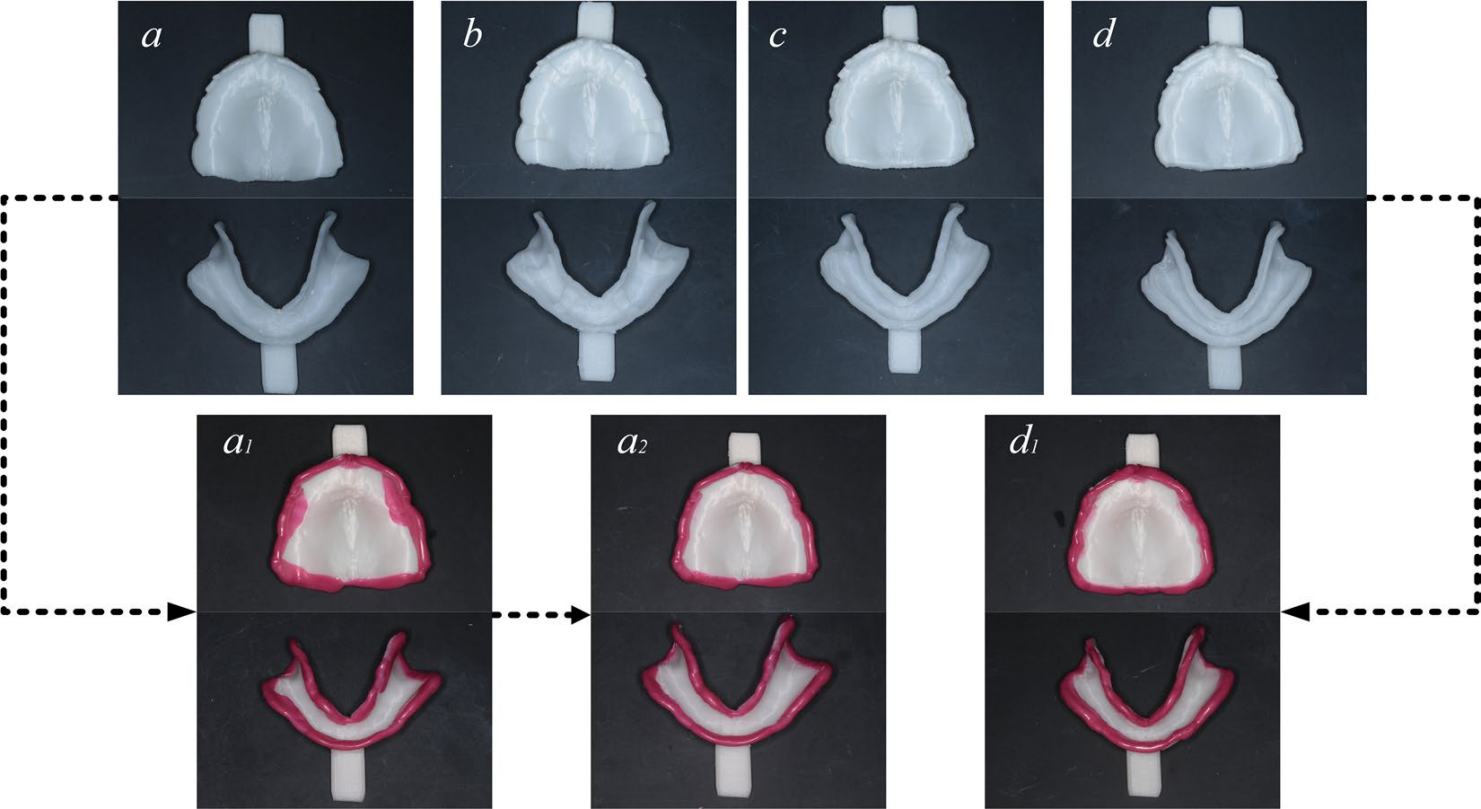
3D-printed custom trays: (**a**) no tissue stop, (**b**) saddle-shaped tissue stop, (**c**) marginal-band tissue stop, and (**d**) inner marginal-band tissue stop; (a1, d1) border mould was made on the tray of (**a**,**d**), a large amount of material covered the tissue surface of the tray; (a2) the material covering the tissue surface was trimmed.

The 3D-printed custom trays are shown in Fig. 4; the 3DP and 3DPIM trays were preborder-moulded with border-moulding wax, while the other two tray types were not. The marginal-band tissue stop was 1 mm in from the margin on the 3DPIM tray, which is convenient for attaching the border-moulding wax. This tissue stop can better support and restrict the material and can prevent it from over-covering the tissue surface. More importantly, the thickness of the border-moulding wax can be easily controlled by the 2-mm high marginal-band support. Saddle-shaped tissue stops on the 3DPS trays and marginal-band tissue stops on the 3DPM trays also provide some support for custom trays.

**Figure 5:**
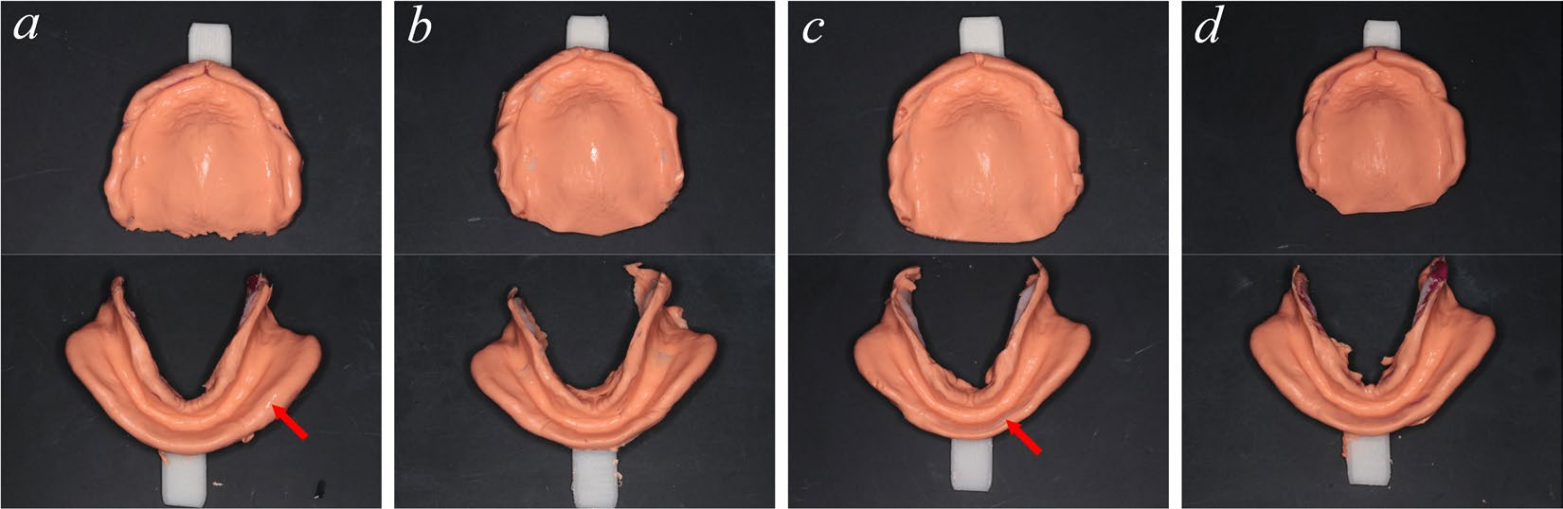
Impressions of edentulous jaws with alginate impression material: (**a**) no tissue stop, (**b**) saddle-shaped tissue stop, (**c**) band-shape margin tissue stop, and (**d**) band-shape inner margin tissue stop; the trays of (**a**,**d**) were preborder-moulded with border-moulding wax.

Four trays made for the same patient are shown in Fig. 5. The 3DPS trays did not achieve an ideal location in the maxillary jaw: a slight left misplacement of the tray led to a wider buccal margin in the left upper posterior impression and a narrower one on the other side. The location effect was better in the mandibular jaw with an appropriate width and a suitable impression extension. The 3DPM trays also achieved an ideal effect, but the lack of border moulding resulted in obvious bubbles in the border seal areas that seriously affected the impression quality. In addition, the area of the tissue stop was too small and obvious excessive pressure could be produced in some areas (arrow on Fig. 5c). The 3DP and 3DPIM custom trays obtained the ideal margin morphology because of the preborder moulding, which produces a good seal and relief effect and allows the impression materials to apply uniform pressure on the edentulous jaw to reduce the local overpressure. However, the thickness of the border-moulding wax could not be effectively controlled in the 3DP trays and led to a non-uniform thickness of the final impression margin (arrow on Fig. 5a). Conversely, the margin width of the impression taken by the 3DPIM tray was uniform owing to the effective positioning of the inner marginal-band tissue stop.

### Impression precision

**Figure 6:**
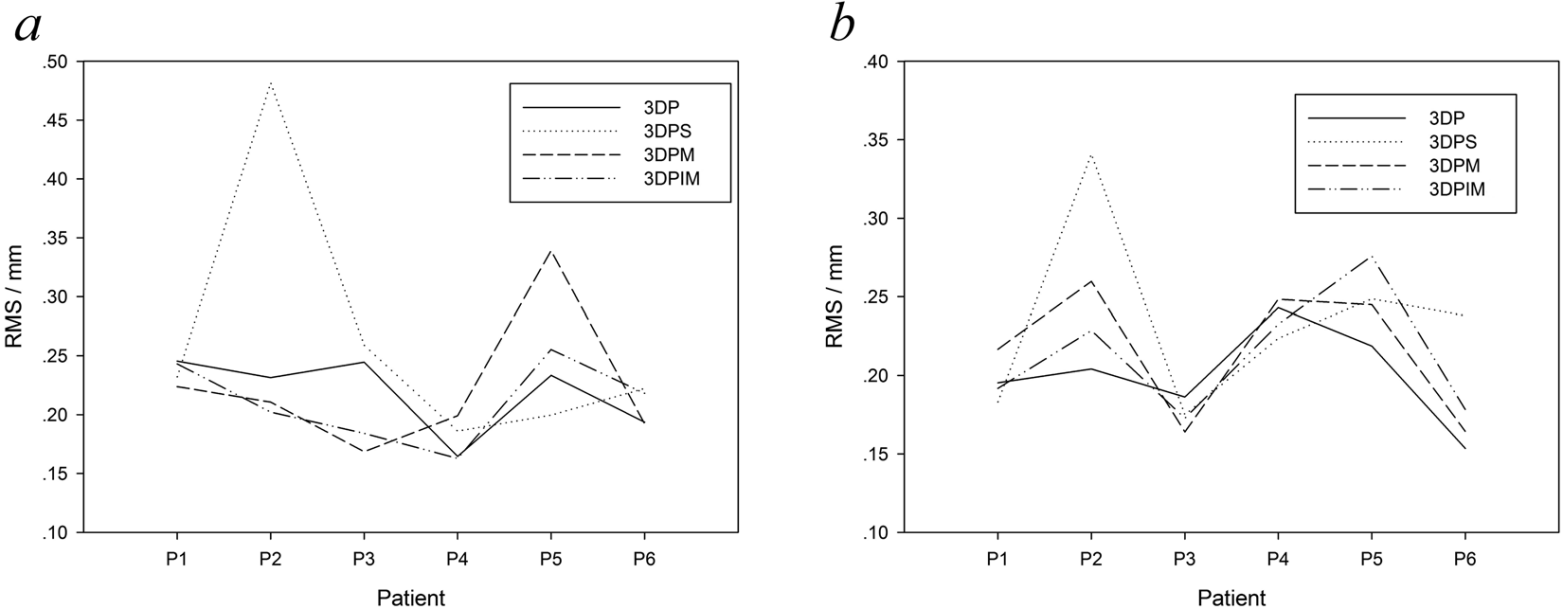
Impressions taken from complete dentures were used as reference data to evaluate the deviations in the impressions taken with the digitised custom trays; (**a**) maxilla and (**b**) mandible.

The deviation value (RMS value) between the four digitised custom tray impressions and the reference impressions from the complete denture are shown in Fig. 6, Tables 1 and 2. For the mandibular impression data, the K-W test result was greater than 0.05, indicating that the data were normally distributed. A randomized block test was conducted, and the P value was 0.308 (>0.05), indicating no significant differences between the impressions from the four mandibular tray sets. For the maxillary impression data, the K-W test showed that the 3DPS and 3DPM groups were not normally distributed. Therefore, a nonparametric Friedman test was used, and the P value was 0.615 (>0.05), indicating no significant differences between the impressions from the four maxillary tray sets. Since denture impressions are considered the most suitable impression, the smaller the RMS value, the closer the impression is to the suitable impression. For the mean value of the RMS, the minimum value of the maxillary impression occurred in the 3DPIM group, followed by the 3DP group, while that of the mandibular impression occurred in the 3DP and 3DPIM groups. Among the six patients in this experiment, the 3DP and 3DPIM groups generally achieved better impression accuracy than did the other two groups. Furthermore, each patient’s maxillary impression results showed that the 3DPIM trays achieved better impression accuracy, while the 3DPS trays deviated greatly for patient “P2”. This was similar for the mandibular impressions as well, as the impression accuracies from the 3DPM, 3DPIM and 3DP trays were similar for all patients, while the 3DPS trays deviated greatly deviation for patients “P2” and “P6”.

**Table 1:** Friedman Test for impression precision of the upper jaw.

Group	Mean ± SD (mm)	Mean Rank	Test Statistics			
			N	Chi-Square	df	P
3DP	0.22 ± 0.03	2.67	6	1.8	3	0.615 > 0.05
3DPS	0.26 ± 0.11	2.17				
3DPM	0.22 ± 0.03	2.17				
3DPIM	0.21 ± 0.04	3.00

**Table 2:** Randomized block test for impression precision of the lower jaw.

Group	Mean ± SD (mm)	Test Statistics					
		Source	Type III Sum of Squares	df	Mean Square	F	P
3DP	0.20 ± 0.03	group	0.004	3	0.001	1.311	0.308 > 0.05
3DPS	0.24 ± 0.03						
3DPM	0.22 ± 0.01	patient	0.025	5	0.005	5.405	0.005 < 0.05
3DPIM	0.21 ± 0.04						

### Margin extension

**Figure 7:**
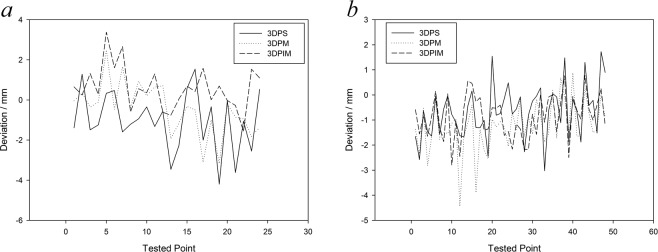
Impressions taken with the preborder-moulded 3DP trays were used as reference data to evaluate the deviation in the margin extensions of the impressions taken with the digitised custom trays; (**a**) values of 24 maxillary sites from six patients and (**b**) values of 48 mandibular sites from six patients.

The impression extensions for the 3DPS, 3DPM and 3DPIM custom trays are shown in Fig. 7, Tables 3 and 4, where the impressions taken by the preborder-moulded 3DP trays were set as the reference. With zero as the reference line, positive values indicated that the extension distance was greater than that of the 3DP trays, while negative values indicated that the mean was lower. The absolute values were statistically analysed for all data. The K-W test showed that the 3DPS and 3DPIM groups for the upper jaw and the 3DPM group for the lower jaw did not satisfy the normality criteria. Thus, the Friedman test was used to evaluate the differences in each group. The results showed that the margin extension of the maxillary impressions from the three custom trays differed significantly (P < 0.05), while no differences were found in the mandibular impressions. Furthermore, since the margin extension of the 3DP group was considered the most suitable margin extension, the closer the measurement to 0, the more suitable the margin extension. As shown in Table 3 and Fig. 7, measurements from 24 maxillary sites from six patients showed that the 3DPIM tray extensions were greater, while those of the 3DPS trays were lower than those of the 3DP trays. The 3DPM trays obtained a greater extension than did the 3DP trays in patients 1–3 but were lower in patients 4–6. The maximum value of the negative deviation occurred in the 3DPS trays, which was 4 mm shorter than that of the 3DP trays. To analyse the mandibular impression extensions, four teeth sites were measured, and data from 48 sites were obtained from six patients. The 3DPS, 3DPM and 3DPIM tray extensions were less than that of the 3DP trays. The fluctuation in deviation values at test points on the impressions from the 3DPIM trays were lower than those of the 3DPS or 3DPM trays. The maximum value of the negative deviations appeared in the 3DPM trays and were at least 4 mm shorter than those in the 3DP trays.

**Table 3:** Mean value and standard deviation of the margin extensions for each group (compared with the 3DP group).

Jaw	Characteristic	Mean ± SD (mm)		
		3DPS	3DPM	3DPIM
Upper	All value	−1.04 ± 1.50	−0.16 ± 1.10	0.61 ± 1.10
	Absolute value	1.43 ± 1.11	0.86 ± 0.68	0.93 ± 0.83
	Positive value	0.79 ± 0.50	1.00 ± 0.81	1.02 ± 0.31
	Negative value	−1.65 ± 1.18	−0.79 ± 0.62	−0.64 ± 0.53
Lower	All value	−0.37 ± 1.06	−1.25 ± 1.01	−0.87 ± 0.88
	Absolute value	1.00 ± 0.76	1.33 ± 0.89	0.99 ± 0.73
	Positive value	0.88 ± 0.65	0.41 ± 0.36	0.34 ± 0.31
	Negative value	−1.02 ± 0.61	−1.44 ± 0.88	−1.15 ± 0.72

**Table 4:** Friedman Test for the absolute value of the margin extension.

Jaw	Mean Rank			Test Statistics			
	3DPS	3DPM	3DPIM	N	Chi-Square	df	P
Upper	1.88	2.31	1.81	48	7.125	2	0.028 < 0.05
Lower	2.4	1.65	1.95	20	5.70	2	0.058 > 0.05

3DP: 3D-printed trays without tissue stop; 3DPS: 3D-printed trays with saddle-shaped tissue stops; 3DPM: 3D-printed trays with marginal-band tissue stops; 3DPIM: 3D-printed trays with inner marginal-band tissue stops.

## Conclusion

Because the sample size was small, significant differences were found only in the margin extension of the upper jaw. However, in terms of mean value and operation experience, the 3DPIM custom tray with the inner marginal-band tissue stop improved the impressions’ accuracy and extension and facilitated the preborder-moulding process.

## Discussion

Owing to the application of CAD and 3D printing technology in dentistry, we can design high-quality tissue stops for custom trays to assist their precise placement in a patient’s mouth to obtain a uniform thickness and suitable impression extension^8^. In this study, we designed saddle-shaped tissue stops (3DPS), marginal-band tissue stops (3DPM) and inner marginal-band tissue stops (3DPIM). Saddle-shaped tissue stops are located at the top of the alveolar ridge and extend 3–5 mm to the slope of the alveolar ridge in the buccal and lingual directions, thereby terminating in the vertical and buccal-lingual directions when the tray is placed. When the trays are placed slightly unevenly, the tissue stop will slide along the buccal or lingual slopes of the alveolar ridge, and finally ride across on the top of the alveolar ridge to achieve its location and support. However, if the initial position deviates too much, the entire tissue stop may be completely turned to one side of the alveolar ridge and cannot be correctly positioned. The marginal-band tissue stop in this study formed a circular support near the custom tray margins, but the function differed from that used in the upper or lower jaw. For the lower jaw, the marginal-band tissue stops in the labial/buccal or lingual side provide both buccal and lingual constraint, respectively. For the upper jaw, the marginal-band tissue stop can effectively prevent backward displacement and provides vertical support in the postdam area but lacks forward constraint and vertical support in the anterior area. Thus, when taking the upper impression, appropriate backward and upward pressure must be applied on the custom tray so that the margin band-shaped tissue stop completely fits the mucosal surface to form the margin sealing. When the tray is placed, the impression material cannot be easily squeezed out even if continually pressed, which can terminate the custom tray in the vertical direction. Consequently, in this experiment, the margin band-shaped tissue stop was better than the saddle-shaped tissue stop and was more uniform with greater impression accuracy.

In this study, the functional impression from the final complete denture was set as a reference to evaluate the accuracy of each tray’s impression. Although the complete denture was manufactured based on the impression taken by the 3DP custom tray, the impression taken from the denture represents the functional state of the patient’s oral mucosa, which differs from the impression taken with the 3DP custom tray. When the patient chews food using the complete denture, the primary stress-bearing area at the alveolar ridge crest bears the most pressure, while the border seal area and the relief area bear the least pressure^15^. Therefore, the non-uniform mucosal deformation will cause the dimensional morphology to deviate from the initial nonfunctional state^15,16^, and this deviation will result in tenderness from the denture if the impression was taken in the nonfunctional state. Due to preborder moulding in the 3DP and 3DPIM custom trays, the impression material is difficult to squeeze out because of the border-sealing effect and therefore produces an appropriate pressure on the mucosa. This pressure is similar to the functional pressure, so the impression morphology may be closer to that of the functional state. The marginal-band tissue stop of the 3DPM custom tray has a similar effect on border sealing, but due to a possible error in the primary impression and the 3D printing process, a satisfactory sealing effect is difficult to guarantee. The 3DPS custom tray cannot form “border-sealing pressure” because it lacks preborder moulding. The saddle-shape tissue stops directly contact the alveolar ridge, which may cause excessive deformation in the contact area, while the pressure in the noncontact area is insufficient. Because of the limited sample size in this study, no significant differences occurred in the RMS, which is an index of the impression precision between the four custom trays. However, the 3DP and 3DPIM trays with prebolder-moulding offered better results in some cases (Fig. 6).

Appropriate extension is important to denture retention. Without hindering the functional activities of the surrounding tissue, the edges of a complete denture base should be fully stretched and possess suitable thickness and shape. These characteristics can maximize the denture base area to maintain close contact with the surrounding soft tissues to form a good border seal^17^. Before the impression is taken, preborder moulding on the custom tray contributes to a proper extension range. Without the preborder moulding, the 3DPS and 3DPM trays easily formed insufficient extensions and were even 3–4 mm short at some custom sites, which could reduce complete denture retention. The 3DPIM tray is beneficial for attaching the border-moulding wax and restricts material overlay on tissue surfaces, thus improving the convenience of the procedure. Additionally, the thickness of the border moulding can be easily controlled by the marginal-band support, thereby ensuring an appropriate marginal thickness in the complete denture base.

The results of this experiment showed that border moulding of the custom tray helped obtain a better extension before taking the final impression. The 3DPIM custom trays have obvious advantages: the marginal-band support facilitates attaching the border-moulding wax, limits the material coverage on the tissue surface, and controls the border moulding thickness so the custom tray can be placed accurately. Therefore, a custom tray with an inner marginal-band tissue stop can be used to improve margin morphology and impression accuracy.
